# A Case of Ischemic Pancreatitis After Acute Aortic Dissection

**DOI:** 10.7759/cureus.79075

**Published:** 2025-02-16

**Authors:** Misaki Fujieda, Takushi Santanda, Ayami Sato, Ryota Hara, Yuichi Nakamura, Syunsuke Shibata, Manabu Yamasaki

**Affiliations:** 1 Department of Critical Care Medicine, Itabashi Chuo Medical Center, Itabashi, JPN; 2 Department of Cardiovascular Surgery, Itabashi Chuo Medical Center, Itabahsi, JPN; 3 Department of Cardiovascular Surgery, Itabashi Chuo Medical Center, Itabashi, JPN

**Keywords:** acute aortic dissection, celiac artery dissection, contrast-enhanced computed tomography, intestinal ischemia, ischemic pancreatitis, postoperative complications, splenic infarction

## Abstract

A 52-year-old man with hypertension presented with sudden-onset lower back pain and numbness in both lower limbs. Imaging revealed a Stanford type A aortic dissection extending from the ascending aorta to the left common iliac artery, with compression of the celiac artery and partial thrombosis of the superior and inferior mesenteric arteries. The patient underwent ascending aortic replacement surgery. On postoperative day 3, he developed intestinal ischemia, requiring a subtotal colectomy. By postoperative day 10, he developed fever and hypotension, and subsequent imaging revealed ischemic pancreatitis localized to the pancreatic body. This was attributed to celiac artery stenosis due to false lumen compression and superior mesenteric artery dissection. He gradually recovered with conservative management, including fluid therapy and percutaneous cyst drainage. This case highlights the importance of recognizing ischemic pancreatitis as a delayed complication of aortic dissection, particularly in cases involving impaired visceral blood flow.

## Introduction

Acute aortic dissection is a life-threatening condition that can lead to various ischemic complications due to impaired blood flow in the major abdominal arteries. Among these, intestinal ischemia is a well-documented and severe complication with a high mortality rate. According to data from the International Registry of Acute Aortic Dissection (IRAD), the in-hospital mortality rates for patients with mesenteric malperfusion who underwent medical, endovascular, surgical, and hybrid therapies were reported to be 95.2%, 72.7%, and 41.7%, respectively [1]. In contrast, pancreatic ischemia is a rare and often overlooked complication. Its rarity in the context of aortic dissection is likely due to the pancreas’ dual blood supply, with the head receiving perfusion from both the celiac artery and the superior mesenteric artery [2]. Here, we report a rare case of acute aortic dissection complicated by both intestinal ischemia and ischemia of the pancreatic body. This case underscores the need to recognize pancreatic ischemia as an underappreciated but potentially significant complication of aortic dissection.

## Case presentation

A 52-year-old man with a history of hypertension was transported to the hospital by ambulance due to sudden-onset lower back pain followed by numbness in both lower limbs. The lower back pain was clearly of aortic origin and was accompanied by progressively worsening lower abdominal pain. Upon arrival at the hospital, his vital signs were clear consciousness, blood pressure 172/64 mmHg, heart rate 66 bpm, and SpO2 97% on ambient air. He did not exhibit gross limb paralysis. The laboratory test results during the clinical course are summarized in Table 1.

**Table 1:** Laboratory test results during the clinical course. eGFR, estimated Glomerular Filtration Rate

Laboratory tests	On admission	Day 3	Day 10	Reference ranges
White blood cell (10^9^/L)	21.5	6.6	31.7	3.3-8.6
Hemoglobin (g/dL)	13.5	9.5	9.7	13.7-16.8
Platelets (10^9^/L)	13.5	7.8	41.8	15.8-34.8
CRP (mg/dL)	0.05	33.39	13.75	0-0.14
Blood urea nitrogen (mg/dL)	25.3	58.3	95.6	8.0-23.0
Creatinine (mg/dL)	1.31	6.66	8.84	0.61-1.08
eGFR (mL/minute/1.73 m^2^)	46.5	7.8	5.8	60-
Aspartate aminotransferase (U/L)	23	1,138	54	8-40
Alanine transaminase (U/L)	21	89	10	5-45
Total bilirubin (mg/dL)	0.9	2.5	5.4	0.3-1.2
Alkaline phosphatase (U/L)	72	63	162	38-113
Lactate dehydrogenase (U/L)	313	1,337	1,081	124-222
Creatine kinase (U/L)	196	5,756	442	55-250
Amylase (U/L)	83		233	44-132
Sodium (mmol/L)	139	142	139	146-147
Potassium (mmol/L)	2.8	4.7	4.9	3.5-5.0
Chloride (mmol/L)	104	101	101	98-108

**Figure 1:**
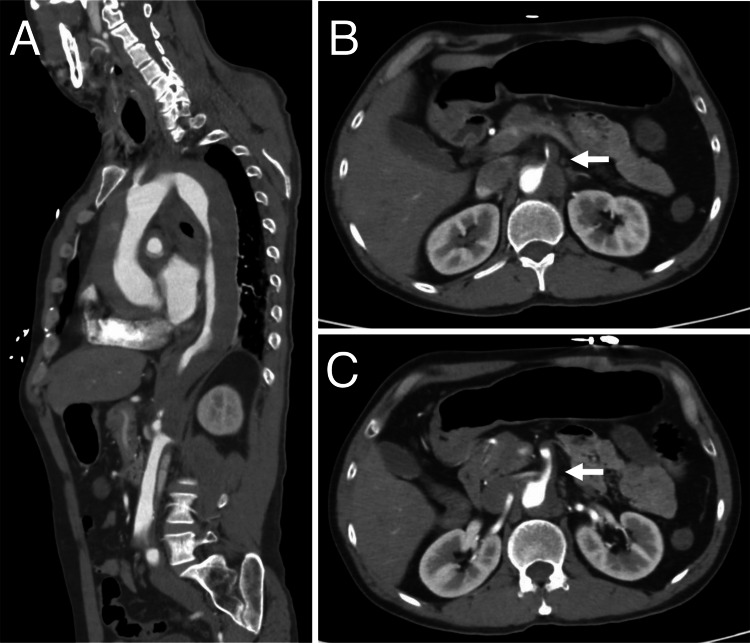
Dynamic contrast-enhanced CT revealing a Stanford type A aortic dissection. A 52-year-old man was transported to the hospital by ambulance with acute back pain as his primary complaint. The sagittal image (A) from the dynamic contrast-enhanced CT scan reveals a Stanford type A aortic dissection. The dissection extends to the celiac artery (B) and superior mesenteric artery (C), with the true lumen compressed by the false lumen.

Dynamic contrast-enhanced computed tomography (CT) revealed a Stanford type A aortic dissection, with a false lumen extending from the ascending aorta to the left common iliac artery (Figure 1).

The celiac artery (CA) was dissected, and the true lumen was compressed by the false lumen. At the same time, partial thrombosis was observed in the superior mesenteric artery (SMA) and inferior mesenteric artery (IMA). On the same day, the patient underwent ascending aortic replacement surgery (J-graft 26 mm, hypothermic circulatory arrest time 41 minutes, and extracorporeal circulation time 184 minutes).

On the third day of hospitalization, he developed mucous-bloody stools, and blood tests revealed elevated creatine kinase (CK) (1,337 U/L) and lactate dehydrogenase (LDH) (5,756 U/L). Dynamic contrast-enhanced CT was performed to evaluate possible intestinal ischemia, revealing poor contrast enhancement from the transverse colon to the rectum, along with splenic infarction. The narrowing of the IMA had progressed, whereas the narrowing of the SMA and CA had improved. A subtotal colectomy from the transverse colon to the rectum with colostomy formation was performed for the diagnosis of colon necrosis secondary to aortic dissection. Intraoperative findings confirmed extensive necrosis extending from the mid-transverse colon to the rectum, with evidence of mucosal blood flow impairment at the rectal stump.

**Figure 2:**
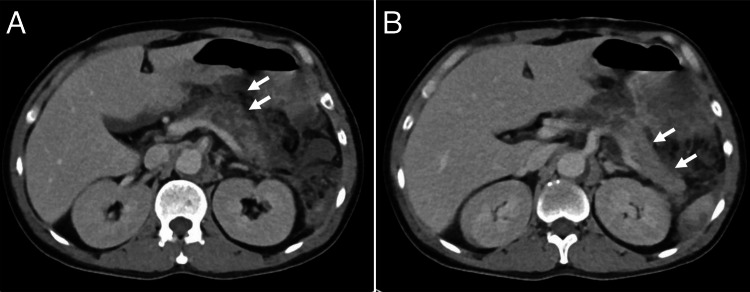
Ischemic pancreatitis of the pancreatic body caused by aortic dissection. An axial image from the contrast-enhanced CT in the equilibrium phase shows a poorly contrasted area in the pancreatic body, along with the increased density of the surrounding fatty tissue, consistent with acute pancreatitis (A). In the pancreatic tail, although the surrounding fatty tissue exhibits increased density, contrast enhancement is preserved (B).

On the fifth day of hospitalization, the patient developed a fever and a heightened inflammatory response, followed by hypotension on the tenth day. Contrast-enhanced CT was performed to reassess intestinal ischemia and identify the source of hemodynamic instability, revealing an enlarged, poorly contrasted area in the pancreatic body (Figure 2).

Serum amylase levels measured after CT were elevated at 233 U/L, leading to a diagnosis of ischemic acute pancreatitis. The patient was managed with appropriate fluid resuscitation and enteral nutrition. On the 14th day of hospitalization, a follow-up contrast-enhanced CT revealed an encapsulated pancreatic cyst, which was treated with percutaneous drainage. Subsequent conservative management led to gradual resolution of the patient's clinical symptoms and inflammatory response. Five months after admission, he was transferred to a rehabilitation hospital.

## Discussion

This case was notable for the development of intestinal ischemia and ischemic pancreatitis following aortic dissection. Generally, the pancreas is considered relatively resistant to ischemic injury due to its dual blood supply. Specifically, the pancreatic head and uncinate process receive blood from arterial arcades formed by branches of the CA and SMA, while the pancreatic body and tail are supplied by arcades formed by branches of the splenic artery. Although ischemic pancreatitis is rare, various etiologies have been reported, including aortic dissection, shock [3], abdominal aortic aneurysm [4], cardiovascular surgery [5], transarterial chemoembolization (TACE) [6], intra-aortic balloon pump (IABP) placement [7], and cocaine use [8]. Umeda et al. reported a case of simultaneous ischemic pancreatitis and ischemic gastropathy due to occlusion of the splenic artery and left gastric artery following aortic dissection. In that case, pancreatitis developed in the pancreatic body and tail despite the patency of the dorsal pancreatic artery (DPA) and transverse pancreatic artery (TPA). The authors speculated that in addition to splenic artery obstruction, cholesterol crystal embolization secondary to aortic aneurysm rupture may have contributed to ischemic injury in the stomach and pancreas [9].

**Figure 3:**
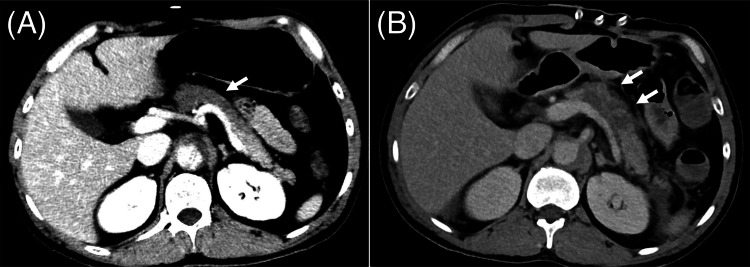
Retrospective review of pancreatic findings. Dynamic contrast-enhanced CT in the arterial phase (A) on the day of admission: When the window level is adjusted, a faint area of poor contrast is visible in the pancreatic body, which later develops into a pseudocyst. Contrast-enhanced CT in the equilibrium phase (B) on the third day: The poor contrast in the pancreatic body is more pronounced and is accompanied by increased density of the surrounding fatty tissue.

In the present case, pancreatitis was confined to the pancreatic body, despite the presence of splenic infarction. This localization was likely due to severe stenosis of the CA caused by compression from the false lumen, as well as obstruction of the interior pancreatic artery, which arises from the SMA. In addition to direct ischemic injury and reperfusion injury, factors such as cardiopulmonary bypass, postoperative systemic changes, and inflammatory cytokine release may have contributed to the development of ischemic pancreatitis [2]. Furthermore, there was no direct or indirect evidence suggesting cholesterol embolism as the underlying cause. Notably, ischemic pancreatitis was diagnosed incidentally during the evaluation of postoperative fever. A retrospective review of the initial contrast-enhanced CT on the day of admission revealed a subtle contrast defect in the pancreatic body, which was overlooked at the time. Additionally, findings suggestive of pancreatitis were present on the CT scan performed on the third day; however, they were overlooked as the physical and imaging findings of intestinal ischemia were more prominent (Figure 3).

As ischemic pancreatitis was not initially suspected, it was not included in the differential diagnosis until the contrast-enhanced CT was performed on the tenth day. The patient experienced persistent postoperative abdominal pain, which was attributed to the surgical wound, making it challenging to recognize the development of new-onset pancreatitis based on clinical findings alone. A review of published case reports in both Japanese and English suggests that the interval between aortic dissection diagnosis and ischemic pancreatitis diagnosis ranges from 12 hours to 15 days (Table 2) [2,9-14].

**Table 2:** Summary of a case report of acute pancreatitis that developed after the diagnosis of aortic dissection. *Conducted 40 days after the onset of aortic dissection. SMA, superior mesenteric artery; CT, computed tomography; CA, celiac artery

Author	Age/Sex	Type of dissection	Initial CT findings of the CA	Initial CT findings of the SMA	Surgery	Time from onset to diagnosis	Time from diagnosis of aortic dissection to diagnosis of acute pancreatitis	Outcome
Hamamoto [2]	47 M	B	Dissected and the splenic artery was occluded	Originates from the true lumen	None	Day 0	12 hours	Survive
Koyama et al. [11]	56 M	B	Proximal obstruction	Originates from the true lumen	None	Day 0	2 days	Survive
Umeda et al. [9]	49 M	B	Dissected	Patent (Details unknown)	None	Day 0	2 days	Survive
Aiba et al. [12]	67 F	A	Dissected	Originates from the true lumen	Ascending aorta replacement ＋ partial arch replacement*	Day 0	4 days	Survive
Oono [13]	60 M	B	Originates from the true lumen	Originates from the true lumen	None	Day 0	7 days	Survive
Jie et al. [10]	52 M	B	Dissected and the splenic artery occluded	Dissected and partially occluded	Thoracic endovascular aortic repair	Day 0	8 days	Survive
Nemoto et al. [14]	71 M	B	Originated from the false lumen	Dissected and partially occluded	None	Day 0	15 days	Survive

This delay is likely due to the time required for pancreatic ischemia to manifest and the difficulty in distinguishing it from symptoms related to aortic dissection. Clinicians should remain vigilant for pancreatic ischemia in cases of CA and SMA malperfusion.

In cases of acute aortic dissection extending to the main abdominal branches or beyond, malperfusion may occur due to compression of the SMA by a false lumen or occlusion of its ostium. According to data from the IRAD, the incidence of mesenteric arterial insufficiency was 3.8%, with in-hospital mortality rates of 95.2%, 72.7%, and 41.7% for patients who received medical, endovascular, surgical, and hybrid treatments, respectively [1]. In contrast, a retrospective study by Wang et al. reported an incidence of acute pancreatitis complicating aortic dissection at 0.29% (6/2,063 cases), with only one fatality, which occurred in a patient who declined treatment [15]. Although reporting bias cannot be ruled out, most reported cases of ischemic pancreatitis following aortic dissection have favorable outcomes, suggesting that its prognosis may be better than that of intestinal ischemia.

## Conclusions

In cases of acute aortic dissection extending to the CA and SMA, delayed ischemic pancreatitis should be considered a potential complication. In patients with vascular malperfusion, such as in this case, where intestinal and abdominal organ ischemia are concerns, abdominal pain may be misattributed to other causes, leading to a delayed diagnosis of ischemic pancreatitis. Therefore, careful follow-up is essential.
